# Systematic review: Safety of surgical male circumcision in context of HIV prevention public health programmes

**DOI:** 10.12688/gatesopenres.13730.1

**Published:** 2022-12-21

**Authors:** Kazuaki Jindai, Tim Farley, Quentin Awori, Anaelia-Siya Temu, Fabian Ndenzako, Julia Samuelson

**Affiliations:** 1 Department of Healthcare Epidemiology, Kyoto University, Kyoto, Japan; 2 Department of Virology, Tohoku University, Sendai, Japan; 3 Sigma3 Services, Nyon, Switzerland; 4 Kenya Medical Research Institute, Nairobi, Kenya; 5 World Health Organization, Geneva, Switzerland; 6 World Health Organization, Regional Office for Africa, Brazzaville, Congo

**Keywords:** voluntary medical male circumcision, male urologic surgical procedures, adverse event, human immunodeficiency virus infection, Africa, adolescent

## Abstract

**Background**: Since the recommendation of voluntary medical male circumcision (VMMC) to reduce the risk of heterosexually acquired HIV, a number of adolescent boys and men in 15 priority countries in Africa have been circumcised. Our primary goal was to identify the incidence of adverse events (AEs) associated with VMMC and to assess the safety profile among adolescent boys 10 – 14 years.

**Methods**: We searched the databases MEDLINE and Embase, WHO, and conference abstracts from 2005 to 2019. The incidence of AEs was estimated by type of AE, size of study and age.

**Results**: We retained 40 studies. Severe and moderate AEs overall were estimated at 0.30 per 100 VMMC clients with wide variability per study type. A higher rate was noted in small and moderate scale programmes and device method research studies compared with larger scale programmes. There was a limited number of studies reporting AEs among younger adolescent boys and they had higher infection-related AEs than those aged 20 years and older. Case studies noted rare AEs such as necrotizing fasciitis, tetanus, and glans injury.

**Conclusions**: AE rates were comparable to those from the randomized controlled trials (RCTs) that led to recommendations and implementation of VMMC in high HIV burden countries, despite being implemented in low resource settings. Clients over time have increasingly included adolescents under the age of 15 years. Studies suggest potentially higher risks in this age group. As VMMC services are sustained, patient safety surveillance systems and promoting a patient safety culture are crucial to identify and mitigate potential harms from medical male circumcision.

## Introduction

Three randomized controlled trial (RCT)s and multiple observational studies demonstrated that medical male circumcision reduced the risk of female-to-male HIV transmission by about 60 % ^1– 3^. This evidence led WHO and Joint United Nations Programme on HIV/AIDS(UNAIDS) to recommend in 2007 that voluntary medical male circumcision (VMMC) be implemented as part of HIV prevention programmes in settings with high HIV prevalence and low male circumcision prevalence ^4^. By the end of 2019 an estimated 27 million adolescent boys and men in 15 priority countries in east and southern Africa had been circumcised and provided with other HIV and STI prevention and health services through public health programmes ^5, 6^. This population is often not reached by health care services.

Medical male circumcision is regarded as a safe procedure when performed by a trained and experienced operator. At the time of the 2007 recommendation, the only systematic compilation on male circumcision safety was from the three well-resourced RCTs of immediate or delayed circumcision, which reported a total of 168 adverse events (AEs) in 5230 surgical procedures (3.2%), though the circumstances in which male circumcision would be provided in VMMC programmes would be expected to differ from the research settings. Hence, care was taken to recognize, mitigate and prevent the risks associated with performing a minor surgical procedure at large scale for long-term prevention against HIV infection in the initial programme implementation plans ^7^. Guidance was developed for training, quality standards and assurance, and safety monitoring for countries and other implementers to apply in their programmes, a large proportion of which were supported by the U.S. President’s Emergency Plan for AIDS Relief (PEPFAR) ^8^. Moreover, as VMMC has been scaled up rapidly in priority countries, clinical cadres other than qualified surgeons or medical officers were crucial to implement programmes in countries with limited surgical human resource capacity. VMMC programmes led by trained mid-level providers with recourse to skilled surgical backup has been shown to be safe and has become a standard practice ^9^. Over the past decade there have been on-going efforts to address barriers and facilitators of uptake of VMMC programmes among adults and adolescents, which have accelerated implementations of VMMC programmes. Hence, it was considered timely to describe systematically the current status of safety, measured by adverse events to further support implementation of safe VMMC for adolescent boys and men in the prioritized countries.

The purpose of this systematic review was to compile all information from the published literature on the safety of surgical male circumcision performed in research studies and VMMC for HIV prevention programmes in African countries, considering, where available, specific surgical methods and client age.

## Methods

Our primary outcome of interest was the incidence of AEs, overall, stratified by type of AE, size of study, and age. A secondary objective was to assess specifically the safety profile among boys ages 10 – 14 years. This review was reported according to the Preferred Reporting Items for Systematic Reviews and Meta-Analyses (PRISMA) guidelines ^10^.

### Literature review and search strategy

A comprehensive MEDLINE and Embase search in June 2019 on general male circumcision safety and methods was supplemented with a search for specific types of AEs and by device-based methods ( *Extended data,* Table 1 ^10^). We restricted inclusion to publications since 2005 and excluded those with no primary data on circumcision safety, which referred exclusively to male circumcision in infants or children, or which reported on the safety of male circumcisions performed for therapeutic reasons.

Titles and abstracts of retained publications were independently assessed by two reviewers to exclude those with no English language abstract and no primary data on safety, efficacy, healing or acceptability of specific circumcision methods in adults or adolescents, and to identify duplicate or overlapping publications. Discrepancies were resolved by discussion including a third reviewer as needed. Additional publications were identified by scanning references in reviews, abstracts, relevant conferences since 2015 (International AIDS Society, Conference on Retroviruses and Opportunistic Infections), and by correspondence with investigators known to be engaged in male circumcision for HIV prevention programmes and assessment of novel male circumcision devices for information on new studies or forthcoming publications. 

Retained studies were grouped into broad categories reflecting their context – well-resourced and closely monitored facilities which implemented the three RCTs of impact of circumcision on HIV incidence, surgical arms in research studies investigating new circumcision devices, pilot VMMC programmes with under 1000 clients, medium sized implementation programmes (more than 1000 but under 10,000 clients), and larger programmes (10,000 clients and more).

### Terminology and outcomes

Circumcision safety was assessed by the number and severity of AEs reported in clients, excluding events which were definitely not related to the circumcision procedure. 

We assessed how each included study reported on the ascertainment of AEs. As we included case reports and case series, we preferred narrative descriptions on the AEs reported in those papers ^11^. The majority of clinical studies followed a common classification of AE severity. That classification was based on definitions adopted in 2013 ^12^ and 2014 by the WHO Technical Advisory Group on Innovations in Male Circumcision ^13^, which were aligned with the terminology of the Global Harmonization Task Force ^14^ and in the Adverse Event Action Guide for VMMC by Surgery or Device ^15^. Moreover, AEs are classified as severe when an AE required intervention by a skilled surgeon ^14^. Moderate AEs included any AE not classified as severe but which required intervention by a trained mid-level health care provider or medication (parenteral, oral or topical). Other AEs were classified as mild ^13^. Similar definitions of AEs were adopted by PEPFAR for notifiable adverse events and in the WHO quality assurance guidance ^16, 17^.

We computed the proportion of clients with AEs and corresponding 95% confidence intervals (CIs) as long as studies included denominator information using Microsoft Excel (ver. 16.65).

## Results

**Figure 1.**
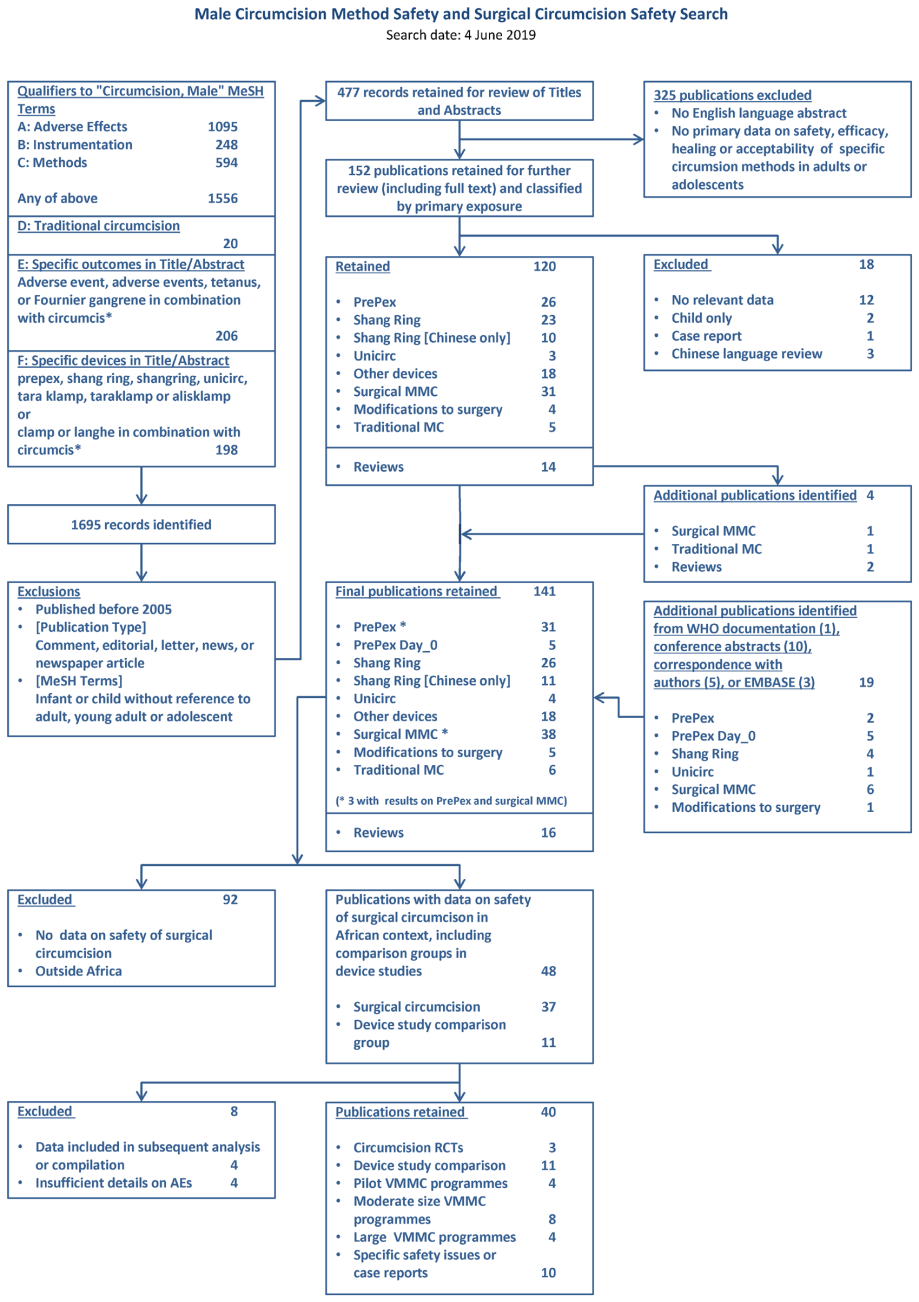
PRISMA flowchart.

**Figure 2.**
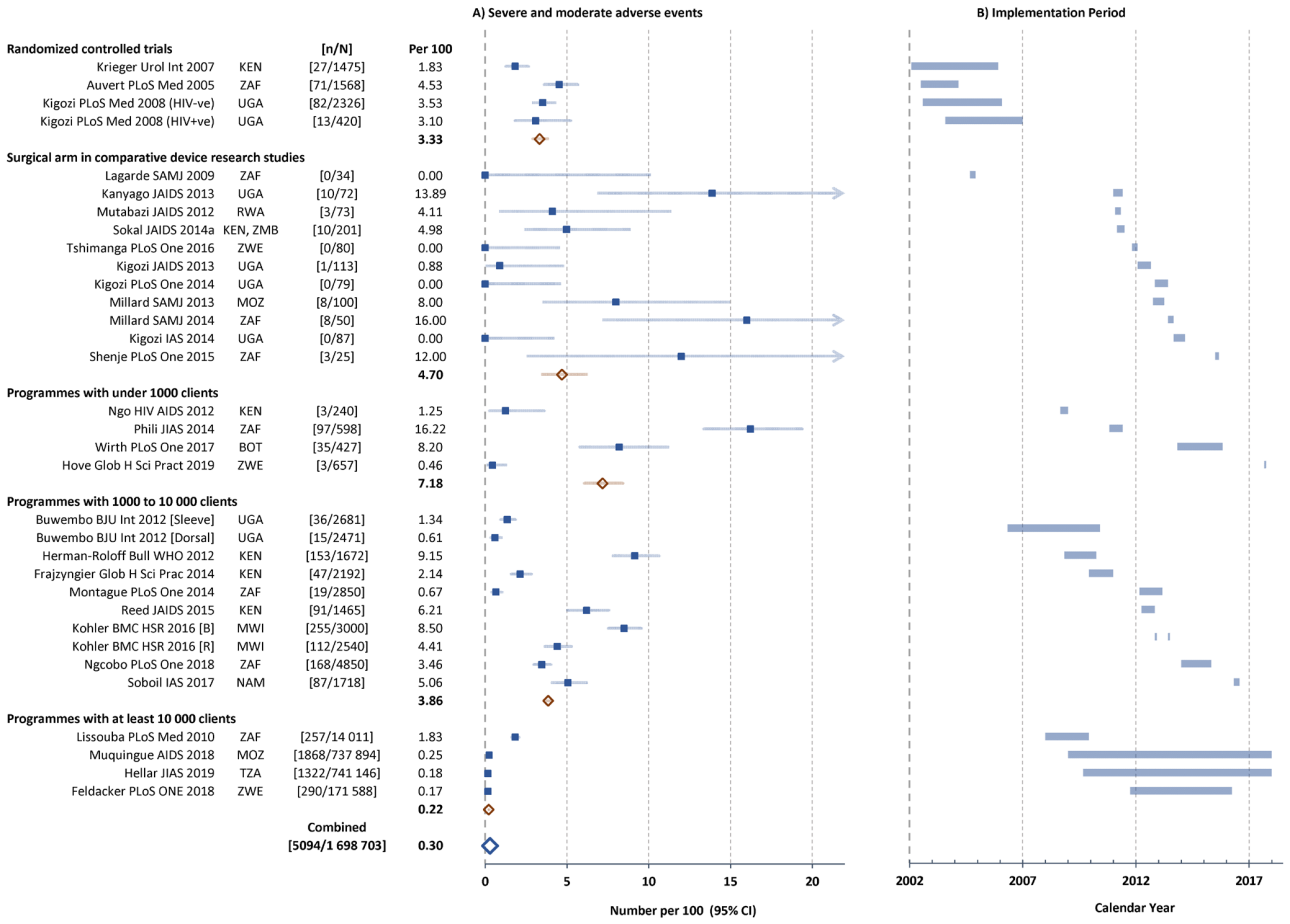
**A**) Severe and moderate adverse events (AEs) in individual studies and **B**) Implementation period of studies reporting circumcision safety (approximate dates of first and last circumcision). Filled squares individual cohorts, open red lozenge all cohorts in subgroup, large open blue lozenge all cohorts combined, lines 95% confidence intervals (truncated line indicated by arrow). ISO-3166 three-letter country codes were use used to represent country. https://www.iso.org/obp/ui/#search/code/

The search identified 1695 records ( Figure 1). After we removed duplicates and reviewed titles and abstracts, 141 were retained for full text review. Restriction to publications with data on the safety of surgical circumcision in Africa left 48, of which eight were excluded because of duplicate or overlapping publication (four) or insufficient data (four) ( *Extended data,* Table 2 ^10^). *Extended data* Table 3 ^10^ summarizes key information from 31 studies with sufficient data to compute proportions of clients with AEs; and also describes country, study design, the type of procedure, the definition of AEs, age of the participants, providers and settings where male circumcision procedures were performed. The time periods covered by the different studies are shown in Figure 2 together with country of implementation. Within each type, studies were ordered chronologically according to the approximate time period when the circumcisions were performed. 

The first three publications ^1, 18, 19^ referred to the safety of surgical circumcision when performed under well-resourced research conditions (RCTs) by trained medical doctors including surgeons. Similarly, 11 studies compared a device-based method to conventional surgical circumcision within well controlled settings, close follow-up and experienced providers (‘Surgical arm’ in comparative device research studies) ^20– 30^.

Within a programme implementation context, 16 studies were undertaken. Many of the ‘smaller-sized studies’ (under 1000 study participants) were part of pilot implementation projects as VMMC was being established within HIV programmes ^31– 34^ or ‘medium sized’ during expansion to new areas or facilities (1000 to 10,000 participants) ^35– 42^. This group included two research studies conducted within VMMC programmes. One study in Uganda started implementation soon after the completion of the Rakai RCT and took the opportunity to compare the dorsal slit and sleeve methods of circumcision which were provided on alternate days of the week ^35^. While not an ideal method of assignment, this ‘randomisation’ was done for logistical simplicity and ensured that client characteristics were reasonably balanced across the two groups. A second publication described the implementation of a quality improvement programme within the VMMC service facility in Malawi, and presented data on AEs during the baseline review of a relatively small number of clinic records and a repeat review conducted six months later ^40^.

The four largest studies (over 10,000 participants) ^43– 46^ cover a similarly mixed variety of settings. One study was a description of the implementation of the post-trial implementation of a VMMC programme within the Orange Farm community, South Africa, which had been the site of the first RCT in South Africa ^1^. Only the total number of Aes were reported with no detail on AE types. The second study was from Mozambique and provided information on the number and types of adverse events in 740,000 circumcision clients ^44^. Data reported to WHO showed that approximately 1.3 million circumcisions were performed in the country over the same period, so this report covered the safety of just over half the total VMMC programme. Similarly, the third study reported from the ZAZIC consortium of partners implementing the Zimbabwe VMMC programme over the period October 2011 to March 2014 and included information on the safety of 171,000 surgical circumcisions, representing approximately 28% of the estimated 610,000 circumcisions performed over the same period ^6, 46^.

The majority of the circumcision providers were specially trained physicians, medical officers (physician assistant equivalent), or mid-level clinical officers or nurses ( *Extended data,* Table 3 ^10^). Experienced urological surgeons performed the circumcisions only in three studies. The settings ranged from minor surgical procedure facilities established for the clinical research studies, general practitioner’s offices equipped for minor surgical procedures, to dedicated high volume fixed or mobile outreach facilities ( *Extended data,* Table 3 ^10^).

### Reported severe and moderate adverse events

Adverse events were considered first by the number of clients with severe and moderate Aes irrespective of type or cause, and then by main type (bleeding, infection, wound dehiscence). Multiple Aes occurring in the same client were classified where possible according to the most severe or with the greatest potential for permanent injury or sequalae if not treated. Apart from the earliest studies, all studies followed the mild-moderate-severe schema first introduced by WHO and PEPFAR in 2009, but there was insufficient detail to distinguish severe and moderate Aes in all studies reliably.

Figure 2 shows the proportion of clients with reported severe or moderate Aes for each study stratified by study type. RCTs showed AE rates between 1.8 and 4.5 per 100 (mean 3.3 per 100). The comparative male circumcision method studies showed considerable variability (range 0 to 13.9 per 100, mean 4.7 per 100). In the comparison between sleeve and dorsal slit methods performed on alternate days, somewhat lower AE rates were noted with the dorsal slit than sleeve method (0.6 compared with 1.3 per 100) ^35^.

In the smaller sized pilot studies implemented as part of HIV prevention through VMMC programmes, the rates were quite variable (range 1.3 to 16.2 per 100, mean 7.2 per 100), while the medium-sized studies were more homogeneous with overall 3.9 per 100. The quality improvement study ^40^ showed rates before and after implementation of the quality assessment – ‘B’ baseline study, ‘R’ repeat survey after 6 months implementation. Overall, the AE rate decreased from 8.5 to 4.4 per 100, mainly due to a reduction in the infection rate (decreased from 5.4 to 1.8 per 100), but small increases were noted with bleeding AEs (increased from 0.3 to 1.5 per 100) and with wound healing AEs (increased from 0.5 to 0.8 per 100).

Programmes with at least 10,000 clients in Mozambique, Tanzania and Zimbabwe reported very low total AE rates (range 0.17 to 0.25 per 100), while the Orange Farm follow-on VMMC programme in the community after the RCT reported 1.83 AEs per 100 ^43^.

**Figure 3.**
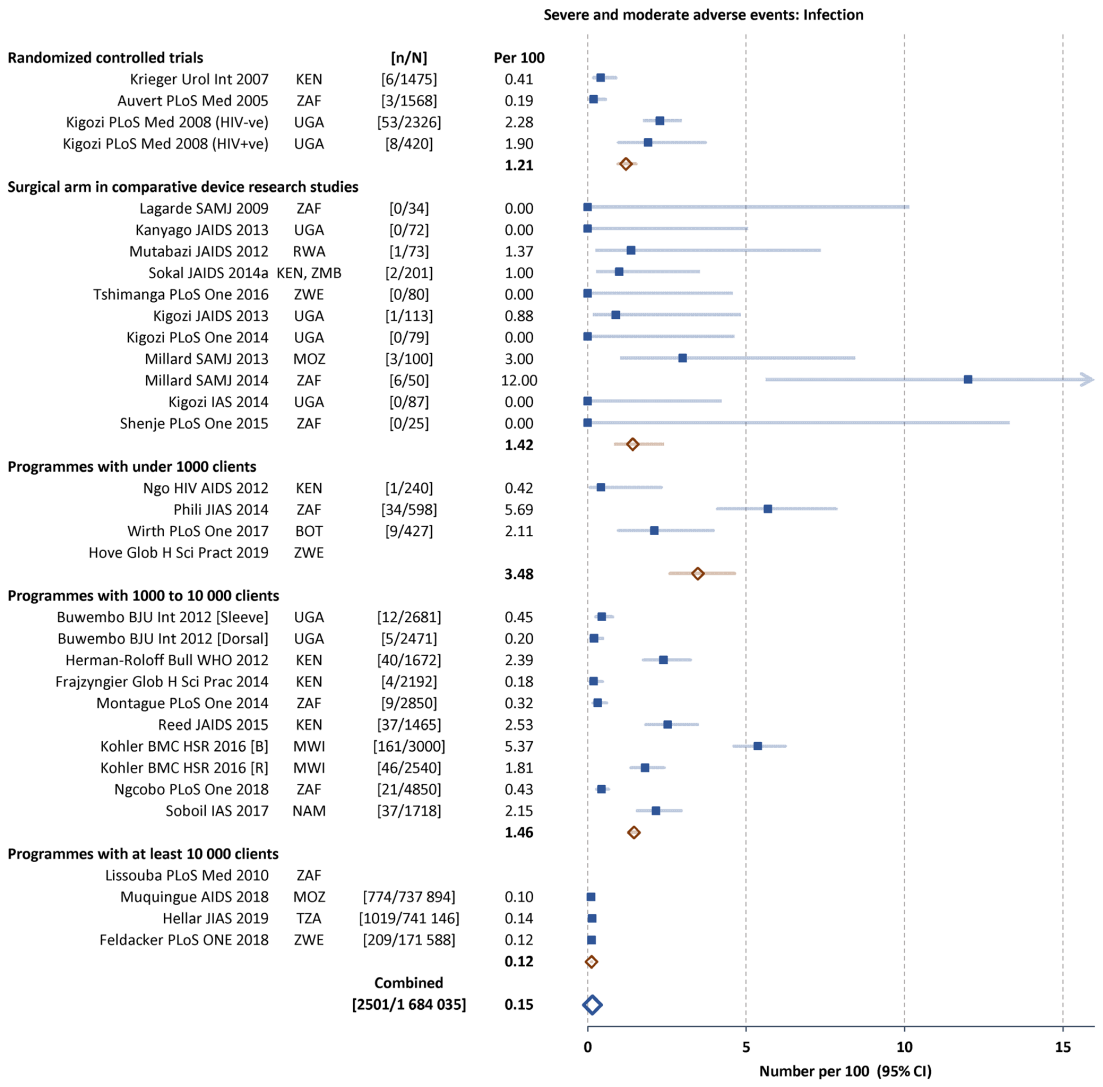
Severe and moderate infection-related adverse events (AEs) in individual studies. Filled squares individual cohorts, open red lozenge all cohorts in subgroup, large open blue lozenge all cohorts combined, lines 95% confidence intervals (truncated line indicated by arrow).

**Figure 4.**
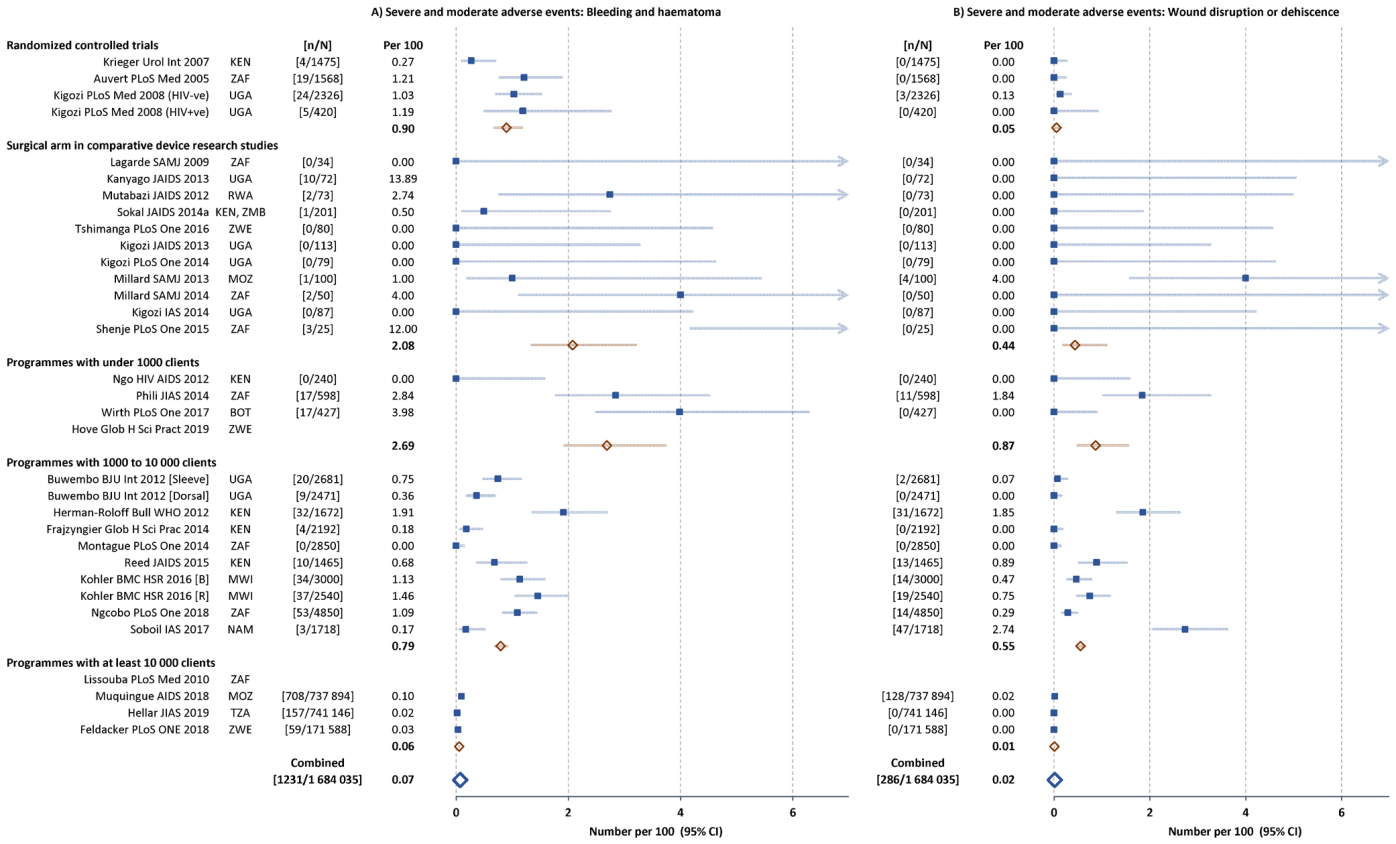
Severe and moderate bleeding- ( **A**) and wound-related ( **B**) adverse events (AEs) in individual studies. Filled squares individual cohorts, open red lozenge all cohorts in subgroup, large open blue lozenge all cohorts combined, lines 95% confidence intervals (truncated line indicated by arrow).

Infection-related AEs represented 50% (2502 of 5114) of all AEs reported, followed by bleeding and haematoma (1237 or 24%) and wound disruption or dehiscence (291 or 6%). The frequency of the main subcategories of AEs were similar across the five types of study ( Figure 3 and Figure 4). In the study comparing dorsal slit and sleeve resection there were fewer AEs of each subcategory with the dorsal slit method.

### Rare adverse events

We also focused on published information on rare AEs considered life-threatening or with long-term consequences. We identified 10 case series or case reports ( *Extended data,* Table 4 ^10^). According to our search strategy, we found serious and rare AEs of the following types and numbers. There were 13 tetanus cases during 2012 through 2016 in five countries among men ages 11–47 years; cases occurred after use of PrePex™ device and conventional surgery, eight of which were adolescents ^47^. Necrotizing fasciitis of the perineum was reported from Kenya ^48^ and Uganda (two cases in 3 years) ^49^. There were 19 bleeding AEs requiring prolonged hospital stay that were reported to the PEPFAR from programmes in multiple countries between 2015 and 2016 (among which five patients developed secondary infection, including one necrotizing fasciitis) ^50^. In Tanzania between October 2014 and September 2016, three glans injury cases were reported ^51^.

### Age specific findings in the age group 10–14 years

Over time, the proportion of VMMC clients shifted to the younger adolescent boys. In the large-scale programme study in Tanzania, over a nine year period, 741,146 clients were circumcised, 51.6% of these clients were in the 10–14 age group ^45^. Similarly, in Mozambique during 2009–2017, where 737,854 sought the service, 52.6% were in the 10–14 age group ^44^.

VMMC clients within the 10–14 year age group were included in 12 of the papers reviewed (one RCT; two in the <1000 category; five in the 1000 – 10,000 category and all four in the >10,000 category). There were varying levels of age disaggregation, type and severity of AEs which limited the compilation of data. More often, the age distribution was given, but not the corresponding breakdown of AEs for each age group. Findings specific to the 10–14 year age group could be found from only two publications on the ZAZIC programme in Zimbabwe.

In the period October 2014 – September 2015 a total of 156 severe and moderate AEs were reported in 44,868 circumcision clients (0.35 per 100, 95% CI 0.3 to 0.41 per 100). The 156 AEs reported represented 25% of total AEs reported over the three-year period. While there was little difference by age group in the incidence of all severe and moderate AEs combined, the incidence of infection-related AEs was approximately 2-fold higher in the 15–19 year age group compared with those 20 years and older, and 3-fold higher in the 10–14 year age group ^52^.

Feldacker and colleagues ^46^ analysed the number and timing of severe and moderate AEs by age group and MC method from the same cohort over the period March 2014 – March 2017 (3 years), but were unable to compute AE rates as the number of procedures performed was not available in sufficient detail over the full time period. They reported a total 617 AEs of which 421 (68%) had complete information on risk factors and 290 had occurred following surgical and 131 following PrePex™ circumcision, which had only been offered to clients aged at least 18 years from April 2014 and aged at least 15 years from July 2016. The majority of the 290 AEs were due to infection (209, 72%) and the remainder were bleeding (59, 20%) or oedema, injury, pain or anaesthesia related (total 22, 8%). The number of AEs by type, days since circumcision and age group showed the largest proportion of infection related AEs were among adolescents with the largest proportion among those 10 – 14 years.

## Discussion

HIV prevention through VMMC remains a priority public health intervention in countries where heterosexually acquired HIV infection is common. We sought to better understand the safety of VMMC since the time period when it was introduced in 2007 and through 2019 when over 27 million men and adolescent boys had been reached. We conducted a systematic review on the prevalence of patient harms, specifically adverse events, associated with surgical male circumcision procedures as recommended for use in public health programmes. 

Data from 40 studies with sufficient data showed severe and moderate AEs occurred overall at 0.3 per 100 VMMC clients, whereas the rate from the original three RCTs was at 3.33 per 100 VMMC clients, which suggests an acceptable level of safety during programme implementation. The lower overall AE rates is dominated by the very low AE rates in the large programmes. Higher rates were noted in the smaller-scale pilot type studies and device method comparative studies than in the larger sized studies which used mostly programme data. This difference in rates possibly reflects closer follow up of participants in smaller-scale pilot type studies and underreporting in large programmes. Accuracy of rates is also affected by correct identification, classification and reporting of AEs, as noted in the quality improvement study. The post-training results showed a shift to lower rates from the pre-training results likely due to improved classification of symptoms associated with normal healing, as well as improved surgical technique.

Infection-related or bleeding-related AEs were also reported with higher occurrence in the smaller-size studies. Tetanus, Fournier’s gangrene, glans injuries and urethral fistula, all serious adverse events, were reported in case series. Such events were rare and most can be prevented. For instance, WHO advised that all patients be assessed for adequate tetanus protection prior to male circumcision procedures, that enhanced attention be given to standard protocols for skin preparation, cleanliness and wound care education ^53^. Fournier's gangrene develops quickly with formidable consequences, and clinical suspicion, urgent care and treatment are the priority.

Regarding adolescents, a limited number of studies, each with method limitations, make interpretation of risk by age group difficult. Younger age adolescents, 10–14 years, were not included in the initial RCTs (18 years and older in two RCTs and 15 years and older in the third). Infection-related AE rates might be higher in the adolescent age group (10–19 years), and more so among younger adolescents, compared with men aged 20 years and older. More recent reports suggested 36 glans injuries and 41 urethral fistulas, nearly always among younger adolescents ^54, 55^.

Glans injury and the risk of urethral fistula can be prevented by using techniques directly visualizing the glans penis and by delaying the procedure until an adolescent boy has a more mature penile anatomy. The value of subregional reporting and response is shown by the reported cases of glans injuries with use of forceps-guided method in young boys. This served as a safety signal that led to the WHO Technical Advisory Group on Innovations in Male Circumcision recommending in 2014 that the method not be used in younger adolescents (particularly boys aged below 15 yeas) ^13^. The reporting at a subregional level of cases of urethral fistula was one reason that WHO recommended VMMC for adults and adolescents aged 15 years and older, seeking to reduce this rare event from conventional surgical methods ^54, 55^. This information comes at a time when a large and increasing proportion of circumcisions performed in national VMMC programmes have been in adolescents, including nearly half in the age group 10 – 14 years (up to 70% for all adolescents) ^56^. Decision makers in national programmes will be determining which age group to focus on as they move to maintain high VMMC coverage levels. Thus, surgical safety is a key consideration on offering VMMC to younger adolescents. To better inform the type, severity and magnitude of adverse events among adolescents, programmes should disaggregate the reported VMMCs and AEs into the smaller band age-groups or by individual ages.

According to the report from PEPFAR supported VMMC programmes, ~85% of patients returned for at least one follow-up visit within 14 days of circumcision ^8^. Moderate or severe AEs were more common among patients who did not come back for a follow-up visit than among patients who did ^39^. Hence, active client follow up is important for more accurate AE reporting and response ^36^.

Our findings also suggest policy implications to further promote the safety of VMMC programmes, knowing that access to safe surgery in general is greatly limited in low-income and lower-middle-income countries, where 90% of people cannot access basic surgical care ^57^.

First, VMMC programmes should enhance patient safety surveillance systems, including the reporting of severe and moderate AEs that occur within 30 days after circumcision procedures. Post-surgical follow-up should be assessed for return contact rates. A positive health-care seeking culture should be enhanced with male populations who tend to have poorer health care seeking behaviours ^58^. Using communication technology and community workers could also be important tactics to ensure post-surgical follow-up ^57^. At the national and sub-regional level, it is important to develop or enhance standardized national and regional surveillance systems for reporting moderate and severe (and serious) AEs including clearly defined protocols and terms of reference for safety monitoring groups. Rare events can best be understood when assessed across the region, so that the number of cases is sufficiently large to identify risks and mitigation factors.

Second, VMMC programmes should work closely with broader patient safety programmes towards an integrated systems approach, supporting development and implementation of patient safety policies at different health administration and care ^59^.

 Third, cultivating a patient safety environment (‘culture of safety’) within a health care system is important to increase the reporting of AEs and to use that information for learning and improving/revising service delivery. Punitive cultures of blaming providers and perhaps patients impede learning and can prevent reporting of safety related incidents and limit effective responses ^59^. A learning culture must be enhanced. Further studies should investigate strategies drawn from other patient safety interventions and participatory learning approaches with community and patient engagement, that build a positive patient safety culture for reporting, learning and responding to moderate and severe AEs in the VMMC settings.

Several limitations of the current study are worth noting. First, AEs can be reported through non-RCT studies including case series/case reports and national surveillance systems that collect AEs during the perioperative period. The quality of such surveillance systems and reporting of *ad hoc* events is likely variable and heterogeneous across countries. Our paper therefore could underestimate moderate and severe AEs; and it may have missed rare but serious AEs that were not detected and reported in the literature within the study period. Secondly, reported AE rates vary. There was wide diversity across studies in how AEs were defined, ascertained, analysed and reported according to setting and intensity of follow-up. This variability makes comparison between studies and programmes difficult. Clients may have a moderate or severe AE but prefer not to return the health service linked with the programme or may manage the complications at home or in another health care facility. Thirdly, we included case reports and case series to identify signals that could potentially further inform about serious AEs. This approach can be inclusive but the risk of bias is high ^11, 60^. Fourthly, variable disaggregation of the AEs either by age-group or type (and severity) makes it challenging to comparatively analyse the study results from these perspectives. As sustaining VMMC for HIV prevention will focus on adolescent boys, such granularity of the data is important.

These limitations notwithstanding, our findings provide insights into the safety of the VMMC programmes that have been implemented over the previous decade. The VMMC programmes over the subregion are generally safe and it is plausible to state that the implementation of VMMC programmes have contributed to improved health with limited harm. Patient safety activities of VMMC programmes need to be sustained and regularly evaluated including as part of programme quality assurance, cultivating a patient safety, and a patient safety surveillance system.

## Data Availability

All data underlying the results are available as part of the article and no additional source data are required. Figshare: Systematic review: Safety of surgical male circumcision in context of HIV prevention public health programmes. https://doi.org/10.6084/m9.figshare.21541392 ^10^. This project contains the following extended data: Reference_gates.docx Supplement_tables.docx Surgical MC Safety Charts & AEs.xlsx Repository: PRISMA checklist and flowchart for Systematic review: Safety of surgical male circumcision in context of HIV prevention public health programmes. https://doi.org/10.6084/m9.figshare.21541392 ^10^. Data are available under the terms of the Creative Commons Attribution 4.0 International license (CC-BY 4.0).
