# Supplementary material for: Systematic review: Safety of surgical male circumcision in context of HIV prevention public health programmes
Source: Gates Open Res. 2023 May 3;6:164. Originally published 2022 Dec 21. [Version 2] doi: 10.12688/gatesopenres.13730.2 (PMC10115943; doi:10.12688/gatesopenres.13730.2)
Supplement: Peer review history

# Peer review: Reviewer response for version 2

**DOI:** 10.21956/gatesopenres.16002.r33139

**Published:** 2023-05-04

**Authors:** Tigistu Adamu Ashengo, Zebedee Mwandi

**Affiliations:** 1 Safe Surgery 2020, Jhpiego, Baltimore, MD, USA; 2 St. Paul M. Medical College, Addis Ababa, Ethiopia; 3 Jhpiego, Nairobi, Kenya

No more comments.

Are the rationale for, and objectives of, the Systematic Review clearly stated?

Yes

Is the statistical analysis and its interpretation appropriate?

Yes

Are sufficient details of the methods and analysis provided to allow replication by others?

Yes

Are the conclusions drawn adequately supported by the results presented in the review?

Partly

Reviewer Expertise:

Surgery, public health, program implementation, implementation research.

We confirm that we have read this submission and believe that we have an appropriate level of expertise to confirm that it is of an acceptable scientific standard.

# Peer review: Reviewer response for version 1

**DOI:** 10.21956/gatesopenres.15018.r32982

**Published:** 2023-04-19

**Authors:** Busisiwe Msimanga-Radebe

**Affiliations:** 1 World Health Organization, Pretoria, South Africa

Thank you for allowing me to review this article. The main aim of this systematic review was to identify the incidence of adverse events (AE) associated with VMMC and stratified according to the type of AE, age and size of the study. The review also assessed the safety profile of boys aged 10-14 years undergoing VMMC. Results showed a higher AE incident in small to medium-scale programmes as well as device methods, compared with larger-scale ones. This was comparable with AE rates from the RTC that showed VMMC can reduce HIV transmission.

 In view of the massive scale-up of the VMMC programme across 15 priority countries, this systematic review addresses a topical issue about the state of safety of the VMMC procedures.

 A well-structured literature search was conducted and a phased flowchart following the Preferred Reporting Items for Systematic Reviews and Meta-Analysis (PRISMA) guidelines was used to illustrate how studies were screened and assessed for eligibility and quality.

 The broad categories of the reviewed studies were well thought through with clearly documented policy implications to promote the safety of VMMC programmes. The authors have outlined the study limitations in detail and have included possible underreporting of the outcome of interest. This will contribute to reducing possible selective reporting

Are the rationale for, and objectives of, the Systematic Review clearly stated?

Yes

Is the statistical analysis and its interpretation appropriate?

Yes

Are sufficient details of the methods and analysis provided to allow replication by others?

Yes

Are the conclusions drawn adequately supported by the results presented in the review?

Yes

Reviewer Expertise:

Public Health specialist with expert experience in VMMC

I confirm that I have read this submission and believe that I have an appropriate level of expertise to confirm that it is of an acceptable scientific standard.

## References

[1] : Adverse events in a large-scale VMMC programme in Tanzania: findings from a case series analysis. *J Int AIDS Soc* .2019;22(7) : 10.1002/jia2.25369 e25369 10.1002/jia2.25369 31368235PMC6669321

# Response

**Published:** 2023-04-26

**Authors:** Kazuaki Jindai

**Affiliations:** Tohoku University, Japan

Thank you very much for your thoughtful comments.

# Peer review: Reviewer response for version 1

**DOI:** 10.21956/gatesopenres.15018.r32815

**Published:** 2023-01-26

**Authors:** Tigistu Adamu Ashengo, Zebedee Mwandi

**Affiliations:** 1 Safe Surgery 2020, Jhpiego, Baltimore, MD, USA; 2 St. Paul M. Medical College, Addis Ababa, Ethiopia; 3 Jhpiego, Nairobi, Kenya

Thank you for the opportunity to review this paper.

 As a systematic review, the reviewers did a decent job identifying relevant articles on Adverse events in VMMC conducted for HIV prevention, and it is a commendable work. I also appreciate the limitations disclosed by the authors. However, there are three areas I think the authors could have explored further to make sure the review provides additional insight to this subject as well as the obvious focus to link it with overall safety culture. 

1. While there are some discussion points given about the lower AE rate in large VMMC implementation programs compared to small pilot studies and RCTs. Which mainly is labeled as under-reporting, while that is an important reason, it would have been an opportune moment to do a possible ‘estimate’ of the adverse event amongst men/boys who were LTFU after VMMC. Earlier abstracts from east Africa (I will try and find and share) indicated high AE rates amongst men who did not return to health facilities but sought AE care in other health facilities, or private clinics away from the clinic where procedures were performed. The systematic review can be of an added value if there is an attempt to find studies and/or program reports that documented not only measured AEs but also captured VMMC related AEs outside the VMMC program, especially for those LTFU from the VMMC program. This would have given a slightly bigger ‘tent’ to capture more AEs related to VMMC.
2. In this systematic review Adverse Events are designated, rightly, as important markers of safety and safety culture. In the title, the discussion and recommendation. It would have further strengthened this argument of safety in the discussion if there was a summary of safety interventions implemented or not implemented vis-à-vis the adverse event rates and observe any association between the two. This is important for the reader because there is an explicit recommendation in the end to implement safety measures to reduce AEs.
3. Related to the above point, if there was any data about the maturity of safety programs/in surgical procedures in any of the locations where the AEs were collected from, would have also given an initial correlation between such practices and overall documentation of AEs in VMMC. I recognize this maybe difficult to do because of the methodological decision to look at papers only on VMMC safety as opposed to safety culture in minor surgical procedures. The authors may look into a follow up review that looks at a slightly broader search objective.

I also shared the paper with my colleague who is overseeing VMMC programs in eastern and southern Africa, Dr. Zebedee Mwandi, who also provided the following feedback. "Overall, I didn’t find anything to worry or correct in my review. The systematic review reinforces the existing literature related to AE in VMMC settings, their prevalence, age at most risk, as well as types/severity. They have highlighted well the limitations to the study, and the recommendations are on point – with emphasis that safety and quality need to be sustained and regularly evaluated as we cultivate culture of patient safety, issues that I totally agree with.”

Are the rationale for, and objectives of, the Systematic Review clearly stated?

Yes

Is the statistical analysis and its interpretation appropriate?

Yes

Are sufficient details of the methods and analysis provided to allow replication by others?

Yes

Are the conclusions drawn adequately supported by the results presented in the review?

Partly

Reviewer Expertise:

Surgery, public health, program implementation, implementation research.

We confirm that we have read this submission and believe that we have an appropriate level of expertise to confirm that it is of an acceptable scientific standard.

# Response

**Published:** 2023-04-26

**Authors:** Kazuaki Jindai

**Affiliations:** Tohoku University, Japan

*Thank you for the opportunity to review this paper.*

*As a systematic review, the reviewers did a decent job identifying relevant articles on Adverse events in VMMC conducted for HIV prevention, and it is a commendable work. I also appreciate the limitations disclosed by the authors. However, there are three areas I think the authors could have explored further to make sure the review provides additional insight to this subject as well as the obvious focus to link it with overall safety culture.*

*1.* *While there are some discussion points given about the lower AE rate in large VMMC implementation programs compared to small pilot studies and RCTs. Which mainly is labeled as under-reporting, while that is an important reason, it would have been an opportune moment to do a possible ‘estimate’ of the adverse event amongst men/boys who were LTFU after VMMC. Earlier abstracts from east Africa (I will try and find and share) indicated high AE rates amongst men who did not return to health facilities but sought AE care in other health facilities, or private clinics away from the clinic where procedures were performed. The systematic review can be of an added value if there is an attempt to find studies and/or program reports that documented not only measured AEs but also captured VMMC related AEs outside the VMMC program, especially for those LTFU from the VMMC program. This would have given a slightly bigger ‘tent’ to capture more AEs related to VMMC.*

 Thank you very much for your thoughtful comment. There is an article assessing the “lost-to-follow-up” that we cited in our manuscript (reference #39), though the study was conducted in 2012 and was small. Regardless, the study does point to the need for programmes to attempt to consider the higher rates amongst LTFU. https://journals.lww.com/jaids/Fulltext/2015/05010/Implementation_and_Operational_Research_.18.aspx.

 Hence, we modified the 6th paragraph in Discussion

 “According to **a 2015** report from **a** PEPFAR supported VMMC programmes, **in 2012**, ~85% of patients returned for at least one follow-up visit within 14 days of circumcision ^8^ . Moderate or severe AEs were more common among **clients** who did not **return** for a follow-up visit than among **clients** who did ^39^ . **Active client**follow up is important for more accurate AE reporting and response ^36^ **The VMMC programmes should be supported to strengthen the VMMC safety surveillance system to identify, at least on a periodic basis,  AEs among those clients who do not return to services even outside the VMMC programmes**.” 

*2.     In this systematic review Adverse Events are designated, rightly, as important markers of safety and safety culture. In the title, the discussion and recommendation. It would have further strengthened this argument of safety in the discussion if there was a summary of safety interventions implemented or not implemented vis-à-vis the adverse event rates and observe any association between the two. This is important for the reader because there is an explicit recommendation in the end to implement safety measures to reduce AEs.*

 Thank you very for this comment. Various documents exist that point to the need for safety surveillance including the WHO UNAIDS AE guidance; the PEPFAR guidance; and country guidance and documents on responses to specific types of AEs. However, we humbly think it would require a separate paper to look at the interventions implemented in response to AE rates and to specific AEs

 Hence, we add the following sentence to 8 ^th^ paragraph in discussion:

 “First, VMMC programmes should enhance patient safety surveillance systems, including the reporting of severe and moderate AEs that occur within 30 days after circumcision procedures. Post-surgical follow-up should be assessed for return contact rates. A positive health-care seeking culture should be enhanced with male populations who tend to have poorer health care seeking behaviours ^58^ . Using communication technology and community workers could also be important tactics to ensure post-surgical follow-up ^57^ . At the national and sub-regional level, it is important to develop or enhance standardized national and regional surveillance systems for reporting moderate and severe (and serious) AEs including clearly defined protocols and terms of reference for safety monitoring groups. Rare events can best be understood when assessed across the region, so that the number of cases is sufficiently large to identify risks and mitigation factors. **Moreover, s** **urveillance of AEs permits to inform relevant responses that could lower higher rates of AEs including for type-specific events (such as tetanus, urethral fistula).** ****“

*3.     Related to the above point, if there was any data about the maturity of safety programs/in surgical procedures in any of the locations where the AEs were collected from, would have also given an initial correlation between such practices and overall documentation of AEs in VMMC. I recognize this maybe difficult to do because of the methodological decision to look at papers only on VMMC safety as opposed to safety culture in minor surgical procedures. The authors may look into a follow up review that looks at a slightly broader search objective.*

 Thanks for your great suggestion. Yes, it would be good to look into such the maturity of the safety culture in relation to the AE rates, however as you note that would be another exercise and paper. 

*I also shared the paper with my colleague who is overseeing VMMC programs in eastern and southern Africa, Dr. Zebedee Mwandi, who also provided the following feedback. "Overall, I didn’t find anything to worry or correct in my review. The systematic review reinforces the existing literature related to AE in VMMC settings, their prevalence, age at most risk, as well as types/severity. They have highlighted well the limitations to the study, and the recommendations are on point – with emphasis that safety and quality need to be sustained and regularly evaluated as we cultivate culture of patient safety, issues that I totally agree with.”*

 Thank you very much to Dr Adamu and also thanks for asking Dr Mwandi for the review and sharing a feedback with us.
